# Tissue‐engineered tracheal implants: Advancements, challenges, and clinical considerations

**DOI:** 10.1002/btm2.10671

**Published:** 2024-04-22

**Authors:** Shixiong Wei, Yiyuan Zhang, Feixiang Luo, Kexing Duan, Mingqian Li, Guoyue Lv

**Affiliations:** ^1^ Department of Hepatobiliary and Pancreatic Surgery, General Surgery Center The First Hospital of Jilin University Changchun China; ^2^ Department of Thoracic Surgery The First Hospital of Jilin University Changchun China

**Keywords:** clinical trial, graft, tissue engineering, tracheal substitute, tracheotomy

## Abstract

Restoration of extensive tracheal damage remains a significant challenge in respiratory medicine, particularly in instances stemming from conditions like infection, congenital anomalies, or stenosis. The trachea, an essential element of the lower respiratory tract, constitutes a fibrocartilaginous tube spanning approximately 10–12 cm in length. It is characterized by 18 ± 2 tracheal cartilages distributed anterolaterally with the dynamic trachealis muscle located posteriorly. While tracheotomy is a common approach for patients with short‐length defects, situations requiring replacement arise when the extent of lesion exceeds 1/2 of the length in adults (or 1/3 in children). Tissue engineering (TE) holds promise in developing biocompatible airway grafts for addressing challenges in tracheal regeneration. Despite the potential, the extensive clinical application of tissue‐engineered tracheal substitutes encounters obstacles, including insufficient revascularization, inadequate re‐epithelialization, suboptimal mechanical properties, and insufficient durability. These limitations have led to limited success in implementing tissue‐engineered tracheal implants in clinical settings. This review provides a comprehensive exploration of historical attempts and lessons learned in the field of tracheal TE, contextualizing the clinical prerequisites and vital criteria for effective tracheal grafts. The manufacturing approaches employed in TE, along with the clinical application of both tissue‐engineered and non‐tissue‐engineered approaches for tracheal reconstruction, are discussed in detail. By offering a holistic view on TE substitutes and their implications for the clinical management of long‐segment tracheal lesions, this review aims to contribute to the understanding and advancement of strategies in this critical area of respiratory medicine.

**Translational Impact Statement**

This review provides a nuanced exploration of historical attempts and lessons learned in tracheal tissue engineering. Addressing critical challenges such as insufficient revascularization and suboptimal mechanical properties, our work offers innovative insights into manufacturing approaches and clinical applications for airway reconstruction. Emphasizing the need for long‐term follow‐up assessments, our research aims to revolutionize the clinical approach to long‐segment tracheal lesions, contributing valuable advancements to the broader field of tissue engineering.

**Abbreviations**
bFGF: basic fibroblast growth factor
DTGs: decellularized tracheal grafts
MSCs: mesenchymal stem cells
PCL: polycaprolactone
PE: polyethylene
PET: polyethylene terephthalate
PLA: polylactic acid
PU: polyurethane
TBM: tracheobronchomalacia
TE: tissue engineering

## 1 INTRODUCTION

Tracheal afflictions characterized by discontinuity, structural weakening, or constriction pose a substantial threat to life. Pathologies affecting the conducting segment of the respiratory tract manifest through diverse clinical conditions, presenting a spectrum of challenges for effective intervention. Notably, the realm of medical intervention encounters a formidable gap in addressing prolonged and extensive tracheal defects.^1^ Presently, thoracic surgical procedures, encompassing tracheotomy and primary end‐to‐end anastomosis, serve as the conventional recourse for managing short‐length tracheal damage. However, cautionary measures are warranted as these interventions are deemed unsuitable for defects surpassing 6‐cm in adults (or 2‐cm in children).^2^, ^3^ When confronted with such scenarios, patients find themselves navigating treatment modalities like tracheal reconstruction, slide tracheoplasty, and innovative artificial substitutes. Nevertheless, the efficacy of these solutions is compromised by complications such as granulation formation and gradual implant weakening over time, posing critical challenges to their sustained performance.^4^, ^5^ Consequently, an imperative has emerged for the investigation of the strategies for facilitating the development of tracheal‐like structures.

Given the pressing clinical demand and ongoing dynamic initiatives, this comprehensive review aims to shed light on historical efforts in tracheal replacement. Furthermore, it aims to delineate how the insights garnered from past experiences have profoundly influenced and guided the current trajectory of research efforts in tissue engineering (TE). The narrative unfolds by providing a contextual understanding of clinical imperatives, offering insights into pertinent epidemiological considerations, and elucidating the physiological intricacies of the functional trachea. In doing so, it outlines the design prerequisites for an efficacious tracheal substitute. Subsequently, an overview of contemporary reconstruction strategies is presented, followed by a synthesis of the pivotal lessons derived from clinical trials involving diverse technological interventions.

## 2 CLINICAL IMPERATIVES IN TRACHEAL REPLACEMENT

Tracheal afflictions in adults encompass a spectrum of etiologies, predominantly stemming from traumatic injuries, benign conditions (notably tracheobronchomalacia, TBM), and various cancer types (Table 1).^6^, ^7^, ^8^, ^9^, ^10^, ^11^, ^12^, ^13^, ^14^, ^15^, ^16^, ^17^, ^18^ Although precise incidence figures are challenging to ascertain, tracheobronchial injuries contribute to 2.5%–3.2% of trauma‐related fatalities and affect 0.6%–2.1% of trauma cases admitted complicated with neck or pulmonary injuries.^6^, ^7^, ^8^, ^9^, ^10^, ^11^ Malignant tumors invading the trachea often necessitate lesion resection and one‐stage structure reconstruction, encompassing primary Tracheal or bronchus malignancies such as adenoid cystic carcinoma and squamous cell carcinoma.^12^, ^13^ TBM, an underreported condition characterized by tracheal cartilage softening, poses another pathological mechanism leading to airway damage.^1^ Moreover, prolonged intubation in intensive care unit patients can result in laryngotracheal injuries resulting from tracheotomies and endotracheal intubation procedures. Post‐intubation airway stenosis occurs in approximately 5%–20% of intubated cases, and recent evidence suggests a potential rise in tracheal stenosis cases among patients recovering from coronavirus disease 2019 who required mechanical ventilation.^15^ In the pediatric population, congenital anomalies, such as birth abnormalities, esophageal atresia, and tracheoesophageal fistulas, present distinct challenges, with tracheal agenesis occurring as a less common anomaly, impacting less than 1/50,000 infants.^14^, ^16^, ^17^, ^18^

**TABLE 1:** Epidemiological landscape of tracheal aberrations.

Clinical pathology	Epidemiology	References
Trauma‐induced tracheal lesions	2.5%–3.2% of fatalities in trauma cases 0.5%–2.0% of individuals with chest or neck injuries due to trauma <1% of planned orotracheal intubations 0.05%–0.37% of instances involving endotracheal intubations	[6, 7, 8, 9, 10, 11]
Tracheal malignancies	<1% of the population <1% of individuals each year	[12, 13]
Aplasia of trachea	<1% in neonates	[14]
TBM	23% of COPD patients 44% of individuals with chronic bronchitis 1% of the total patients undergoing bronchoscopy	[1]
Intubation‐related tracheal stenosis	6%–21% following intubation	[15]
Congenital esophageal obstruction	<1% in neonates	[16, 17]
Tracheoesophageal fistulas	<1% in neonates	[18]

While tracheal stenosis in adults is typically addressed through resection and anastomosis, this approach is less feasible in infants and children, especially when the lesion involves exceeding 50% of the total trachea length.^2^ Attempts to provide support using various stents, comprising biodegradable alternatives and Palmaz stents, have achieved limited success.^19^ The reported incidence of tracheal pathologies may appear relatively low; Nevertheless, factors like pre‐hospital mortality and symptoms resembling other common diseases contribute to the underestimation of their true prevalence.^9^, ^13^ This diagnostic challenge often leads to elevated morbidity and mortality rates, underscoring the critical clinical need for advancements in tracheal regeneration.

Addressing the multifaceted etiology of tracheal pathologies necessitates a thorough consideration of demographic and physiological variables impacting tracheal morphology. Extensive research underscores significant sexual dimorphism in tracheal dimensions, with males consistently exhibiting larger diameters and greater cartilaginous thickness than females. Noteworthy ethnic disparities have also been identified, particularly narrower tracheal dimensions observed among individuals of Asian descent compared to Caucasians and African Americans. Additionally, advancing age introduces distinct alterations in tracheal structure, characterized by decreased cartilaginous density and increased susceptibility to collapse.^1^, ^5^, ^6^

These nuanced demographic insights underscore the imperative for tailored approaches in tracheal TE. Careful consideration of sex‐specific variations in tracheal dimensions holds promise in optimizing surgical outcomes and minimizing postoperative complications. Similarly, integration of ethnic disparities in tracheal anatomy informs the development of precision interventions tailored to diverse patient cohorts. Moreover, addressing age‐related changes in tracheal biomechanics is crucial for ensuring the resilience and durability of tissue‐engineered grafts, particularly in geriatric populations.^3^ Hence, it is paramount that future research endeavors prioritize the investigation of demographic determinants in tracheal TE, thereby advancing the field towards more effective and individualized therapeutic modalities.

## 3 TRACHEAL REPLACEMENT: A COMPREHENSIVE REVIEW OF THE ATTRIBUTES AND DESIGN PREREQUISITES FOR GRAFTS

To engineer tracheal substitutes that faithfully replicate the intricate biological and anatomical characteristics of the trachea, an in‐depth understanding of its complex structure, cellular functions, mechanical properties, and vascularization is imperative. Despite its seemingly straightforward appearance, the trachea conceals a sophisticated composition with challenging features that demand meticulous replication. Functionally, the trachea acts as the conduit connecting the upper and lower respiratory tracts, facilitating the ventilation, humidification, and mucociliary clearance of inspired air. The structural framework of trachea consists of 18 ± 2 hyaline cartilaginous rings formed by the annular ligament and trachealis muscle, it requires precise replication to maintain patency and resist forces during respiration.^20^

The airway mucous membrane, located in the innermost layer of wall, plays a crucial role in mucociliary clearance and serves as the initial barrier against pathogens. Any proposed alternative substitute construct should be highly secure, facilitating the potential regeneration of pseudostratified respiratory epithelium to mitigate the risk of infections.^21^ The fibrocartilaginous layer in the tracheobronchial area, composed of the cricoid anchored by the trachealis muscle, necessitates a framework for tracheal reconstruction that demonstrates lateral stability and longitudinal flexibility. This scaffold should withstand the various forces encountered during normal respiration, coughing, or other physiological processes.^2^ Mechanical properties, including resistance to cough‐induced fractures, are critical for graft durability and preventing airway lumen collapse.^3^

Modeling the intricate mechanical forces acting on the structure of cervical trachea becomes more complex due to abnormal situations such as coughing, forced respiration, and sneezing. Tracheal cartilage exhibits biphasic material behavior, while the trachealis muscle demonstrates hyperelastic characteristics.^22^ Varied testing methods and specimen types in existing studies pose challenges in understanding native tracheal mechanical properties for potential replacement device design.^23^

Vascularization is a paramount consideration for the success of tracheal grafts. The highly segmental blood supply of the native trachea presents challenges for revascularization in replacement scenarios. Past failures involving solid polymer tubes underscore the significance of an effective tracheal blood supply, emphasizing the need for grafts that support revascularization to ensure optimal performance.^24^, ^25^

The ideal tracheal substitute should exhibit lateral rigidity and longitudinal flexibility. Additionally, it must be biocompatible, non‐immunogenic, non‐carcinogenic, non‐toxic, and resistant to erosion, dislocation, and stenosis over time.^26^ Developing tracheal tissue regeneration approaches necessitates creating a suitable 3D environment for respiratory epithelium and hyaline cartilage formation, fostering a vascularized plexus to prevent necrotic tissue, and enabling immune cell infiltration to avoid infections during the regeneration process. Various strategies have been employed to address this significant challenge, with historical significance and promising contemporary approaches discussed in detail in this review, incorporating the latest research findings and advancements.

## 4 TRACHEAL RECONSTRUCTION APPROACHES

The pioneering efforts in airway reconstruction trace back to the late 19th century, with early groundbreaking attempts documented between 1886 and 1899.^27^, ^28^ The initial human application of primary anastomosis took place in 1886, marking a significant milestone.^29^ Subsequent explorations involved diverse grafting materials, such as autogenous skin,^30^ fascial tissue,^31^ and costal cartilage,^32^ along with the introduction of solid materials in prostheses.^33^ Tubular tracheal substitutes have been the focus of extensive investigation, employing various strategies such as cadaveric tissue flaps,^34^ implanted bioprostheses,^35^ tubes derived from intestinal tissue^36^, ^37^ and different types of aortic grafts.^38^ The outcomes, successes, and limitations of approaches mentioned above in clinical trials will be thoroughly examined in the subsequent discussion. Despite notable efforts, the replacement of long segmental tracheal defects has encountered challenges in achieving sustained success in long‐term studies, highlighting substantial limitations in current treatment options. Recent research efforts have consequently shifted towards exploring TE strategies as a promising solution to solve this enduring clinical challenge.

## 5 TE STRATEGY

TE approaches predominantly hinge on utilizing a biomaterial scaffold, cultivating diverse cell types on the scaffold to generate airway‐like structure, and incorporating growth factors.^39^ TE has demonstrated its potential in addressing the regeneration of various tissue types.^40^ Consequently, TE applications in respiratory medicine, particularly in the realm of tracheal tissue replacement, have garnered increased attention. Diverse TE methodologies have yielded promising endeavors in the development of tracheal substitutes. However, a construct capable of simultaneously addressing vascularization, mechanical stability, and re‐epithelialization remains elusive to date.^41^, ^42^ Nevertheless, the explored techniques exhibit substantial promise and have the potential to establish the gold standard in tissue‐engineered tracheal constructs.

### 5.1 3D printing technology

The application of 3D printing in medical research has gained traction due to its capability for rapid fabrication of customized substitutes for structural reconstruction, allowing modulation of properties such as architecture, mechanical characteristics, and degradation rate.^43^ This technology enables the creation of intricate, patient‐specific, multi‐layered designs tailored to individual tracheal features.^44^ Polycaprolactone (PCL), renowned for its exceptional mechanical properties, controllable in vivo degradation, and long‐term stability, has been a predominant material in 3D printing TE applications. Notably, 3D‐printed PCL stents have obtained approval from the United States Food and Drug Administration for emergent use in pediatric operation to address benign lesions.^45^, ^46^ Consequently, PCL has been widely employed in tracheotomy research, producing substitutes that exhibit remarkable resistance to compressive stresses and assist cartilage regeneration.^47^ Despite these advancements, PCL scaffolds face challenges in pre‐clinical studies, triggering an inflammatory reaction that leads to the granulation formation and subsequent lumen stenosis.^48^ Explorations into novel materials for 3D printing, such as polyurethane (PU), have demonstrated adequate mechanical support and facilitated cartilage formation.^49^ Continual endeavors in 3D printing of tracheal scaffolds, employing diverse materials and distinctive tubular designs reveal promising initial outcomes, although limited by single‐cell type technologies, deficiency of mechanical characterization, and inadequate revascularization.^50^

The emergence of 3D printing in clinical study has sparked the exploration of direct cell‐laden biomaterial printing, often referred to as bioprinting. This procedure reveals potential for creating stented grafts that can support various types of cells, ensuring reproducibility and customization for individual patients.^51^ However, challenges arise in bioprinting with cell‐loaded bioinks, as low bioink melting points can lead to suboptimal biomechanical properties, resulting in the formation of weak structures.^52^ To address this challenge, researchers have employed dual‐headed 3D printers to fabricate structurally robust scaffolds. This entails the fusion of thermoplastic polymers with hydrogels, such as alginate, and the combination of collagen type I hydrogel with PCL.^53^ Instances of PCL combined with sodium alginate hydrogels have shown success in animal models, exhibiting re‐vascularization, ciliary epithelial regeneration, and cartilage formation.^54^ Bioprinting holds promise for tracheal substitutes, but addressing challenges such as maintaining cell viability, appropriate temperature profiles, and withstanding mechanical stress during bioink extrusion requires further research.^55^

### 5.2 Electrospinning technology

Electrospinning stands out as a versatile and indispensable technique applicable to both synthetic and natural polymers, allowing the rapid fabrication of customizable multi‐layered 3D constructs. This method utilizes high voltage to propel the selected polymer onto a collector plate, leading to the formation of nanofibrous structures.^56^ Its widespread adoption in respiratory TE arises from its unique capability to generate fibrous scaffolds closely resembling the size‐scale of native respiratory extracellular matrix using various materials such as PCL, PU, polyethylene terephthalate (PET), and polylactic acid (PLA).^57^, ^58^

Numerous animal trials have been conducted to evaluate the efficacy of different electrospun materials for tracheal substitutes, as detailed in Table 2.^59^, ^60^, ^61^, ^62^, ^63^, ^64^, ^65^, ^66^, ^67^, ^68^, ^69^, ^70^, ^71^, ^72^ Notably, findings from several studies indicate a more favorable biocompatible response in scaffolds loaded with cells compared to cell‐free constructs.^79^, ^80^, ^81^ Additionally, material strength assessments through electrospinning have demonstrated a spectrum ranging from mimicking the physical characteristics of the native airway to surpassing them.^82^, ^83^ While electrospinning exhibits substantial promise as a TE approach for airway reconstruction, it is crucial to acknowledge the limited availability of long‐term animal studies in this domain. Further exploration and comprehensive investigations are warranted to establish the enduring viability and success of electrospun tracheal substitutes.

**TABLE 2:** Animal studies on tissue‐engineered tracheal replacement approaches.

Animal	Sample volume	TE Material	Cell type	Experimental Overview	Duration	Result	References
Dog	*n* = 3	Polypropylene mesh combined with irradiated minced dermal layer	Keratinocytes	Isolated stem cell populations from primary cultures of skin epithelial cells were characterized, labeled with PKH26 dye, and subsequently transplanted onto canine tracheas	5‐month	Transplanted skin epithelial stem cells survive for months on the trachea and can transform into tracheal epithelial cells and chondrocytes	[59]
Dog	*n* = 18	Polypropylene (PP) mesh tube strengthened by a PP spiral, coated with a 1% collagen solution, and subsequently freeze‐dried	Autologous PB versus BM aspirate versus BM MSCs	The prosthesis, comprising a polypropylene mesh tube reinforced with a polypropylene spiral and an atelocollagen layer, replaced the cervical tracheas in 18 beagle dogs	6‐month	Bone marrow aspirate and mesenchymal stem cells promote tracheal mucosa regeneration on prosthesis	[60]
Dog	*n* = 10	Porous scaffold composed of a PLGA copolymer, affixed to a prosthesis framework crafted from polypropylene (PP) mesh reinforced with PP rings	Keratinocytes	Implanted in the peritoneal cavity for 1 week, enveloped in the greater omentum to promote prosthesis vascularization. Subsequently, a complete surgical resection and replacement of a thoracic tracheal segment (5 cm) were conducted using the prosthesis	2‐month	Highly biocompatible tracheal prosthesis could prove useful for the reconstruction of large, circumferential tracheal defects	[61]
Dog	*n* = 5	Nitinol frame coated in 3% freeze‐dried collagen	N/A	The synthetic trachea was initially implanted in the pedicled omentum and positioned within the abdominal cavity. After 3 weeks, the omentum‐wrapped synthetic trachea was relocated to the thoracic cavity. Subsequently, a partial resection and reconstruction of the thoracic trachea were performed using the synthetic trachea	18‐month	The innovative nitinol artificial trachea faithfully replicated the physical attributes of the native trachea. Cell engraftment, excellent biocompatibility, and a lifespan of 18 months or more for this artificial trachea have been verified in canine models	[62]
Goat	*n* = 10	PCL scaffold engineered with autologous auricular cartilage cells	Autologous articular chondrocytes	Scaffolds were loaded with 1.2 × 108 chondrocytes within a type I collagen gel and incubated for 1 week. A 3.5 cm segment of the trachea was excised and either regrafted as a control or replaced with the implanted scaffold	3‐month	While bronchoscopy and computed tomography confirmed tracheal narrowing in the experimental group, tissue necrosis was exclusively observed in the control group. Moreover, a promising development of epithelial‐like tissue was noted in the Tissue‐Engineered Tracheas (TETs) post‐transplantation	[63]
Rat	*n* = 10	Synthetic nonwoven mesh	Heterologous chondrocytes	The cell‐seeded mesh, wrapped around a silastic stent, was implanted into nude mice for 4 weeks to promote cartilage formation before addressing the cervical defect	1‐week	Bovine chondrocytes successfully produced cartilage. The lack of vascularization was identified as a significant issue	[64]
Swine	*n* = 5	Decellularized tracheal graft	Chondrocytes and epithelial cells derived from autologous MSCs	The decellularized trachea was seeded with either a single cell type or a combination of both cell types and then implanted into a 6 cm defect	2‐month	The seeding of both epithelial and mesenchymal stem cell‐derived chondrocytes is necessary for optimal graft survival	[65]
Rabbit	*n* = 6	PCL collagen sponge	Chondrocytes	The scaffold underwent an 8‐week culture within an in vitro bioreactor to enhance collagen content and promote chondrocyte expansion before implantation	2‐month	Re‐epithelialization of the respiratory epithelium at the anastomotic sites was observed, accompanied by excessive granulation tissue growth	[66]
Rabbit	*n* = 10	Polypropylene mesh	Hairless epithelium	A three‐stage procedure involves obtaining a hairless epithelial graft, creating a prefabricated neotrachea using a tubed structure, and adapting it to the native trachea, followed by a 4‐week evaluation in animals	4‐week	All prefabricated neotracheas and epithelial grafts exhibited viability, with comparable rigidities, longitudinal elasticities, diameters, and wall thickness to native tracheas	[67]
Rat	*n* = 3	Scaffold‐free tissue‐engineered structure	Human cartilage cells, fibroblasts, umbilical vein endothelial cells, and bone marrow‐derived MSCs	Human cartilage cells, fibroblasts, umbilical vein endothelial cells, and bone marrow‐derived mesenchymal stem cells are formed into spheroids and loaded into a bio‐3D printing system equipped with specialized needles arranged based on 3D configuration data	N/A	The tubes are viable in vivo and the tracheal epithelium and capillaries proliferate	[68]
Sheep	*n* = 8	Electrospun blend of PET and PU reinforced with medical‐grade PC rings	Autologous BM‐MNC	Bone marrow‐derived mononuclear cells (BM‐MNC) were concentrated using gravity‐filtered size exclusion and subsequently vacuum‐seeded onto the scaffold. Following this, a 5 cm segment of the native trachea was isolated and resected along with the seeded graft	N/A	Graft stenosis present in all, encapsulation and infection occurred, and no re‐epithelialization but granulation was observed	[69]
Rabbit	*n* = 20	3D‐printed PCL	Chondrocytes	Two groups were investigated, involving scaffolds cultured in chondrocyte suspension for either 2 or 4 weeks, and subsequently implanted into a 1.6 cm defect for assessment	10‐week	Sufficient mechanical properties were observed with no collapse, but the most common cause of death in both groups (75%, 15/20 animals) was attributed to granulation tissue formation, and re‐epithelialization did not occur	[49]
Rabbit	*n* = 4	3D‐printed PCL	N/A	Four‐axis FDM 3D‐printed scaffolds were explored for tracheal replacement	2‐month	No severe narrowing observed; 4‐axis scaffolds exhibited reduced inflammation and superior mucosal regeneration compared to conventional scaffolds, featuring ciliated epithelium in the lumen	[70]
Swine	*n* = 7	3D‐printed PCL combined with decellularized bovine dermal collagen matrix	N/A	3D‐printed tracheal rings were sutured to a decellularized collagen matrix to create composite grafts, ensuring improved tissue integration and serving as a structural foundation for cellular infiltration and growth	3‐month	Death causes were attributed to pneumonia and airway stenosis from granulation tissue and secretions. After 3 months, grafts showed vascularization and ciliated epithelium, but granulation tissue persisted in all surviving animals	[71]
Rabbit	*n* = 12	3D‐bio‐printed scaffolds composed of PCL and sodium alginate hydrogel	MSCs derived from bone marrow and rabbit epithelial cells	A multi‐layered scaffold composed of PCL and sodium alginate hydrogel was utilized, featuring two layers of PCL for structural support. The three hydrogel layers incorporated either 1 × 107 MSCs or epithelial cells per 10 mL	4‐week	Ciliated epithelial mucosa fully covered all scaffolds, and abundant neo‐vascularization was observed across all groups	[72]
Rabbit	*n* = 7	3D‐printed PCL combined with alginate/collagen type 1 hydrogel loaded with chondrocytes	Chondrocytes	A dual‐headed 3D printer was employed to create a scaffold using PCL and alginate/collagen type 1 hydrogel with incorporated chondrocytes	6‐week	During the surgical procedure, one animal experienced a fatality due to an airway blood clot, while the remaining subjects exhibited respiratory distress. Subsequent examination revealed a substantial stenosis rate of 83.4%	[53]
Rabbit	*n* = 16	3D‐printed PLLA	Chondrocytes	3D‐printed scaffolds, immersed in chondrocyte/hydrogel blend, underwent a 3‐day culture. Pre‐vascularization of scaffolds occurred over 2 weeks before implantation. Two in vivo groups were studied: one without pre‐vascularization and another with 2 weeks of pre‐vascularization.	4 weeks (in vitro for 2 weeks and in vivo for another 2 weeks)	The control group experienced severe complications, with an average survival of 17 ± 7 days post‐op, while in the experimental group, six out of eight animals survived until the 2‐month endpoint. Tracheal stenosis was predominantly observed in the control group	[73]
Rat	*n* = 27	Collagen/P(LLA–CL) fiber electrospun scaffold	RTEC and RTC	A bilayered electrospun scaffold with dense fibers on the inner layer and porous yarns on the outer layer was investigated in three groups: PV (pre‐vascularization), CS (cell seeding), and a control group. RTECs and RTC were seeded into the inner lumen and outer surface, respectively	7‐day	Higher immunological indicators were found in bare and CS (cell‐seeded) scaffolds, while PV (pre‐vascularization) scaffolds showed levels similar to the control. PV and CS scaffolds exhibited in‐growing capillaries, with PV scaffolds displaying a simple ciliated columnar epithelium and a continuous cartilage cell layer. In contrast, CS scaffolds showed only flat epithelium and irregular chondrocytes. Bare scaffolds lacked epithelial cells, with an abundance of inflammatory cells in the submucosa	[74]
Swine	*n* = 6	Decellularized tracheal graft	N/A	Decellularization was conducted using the HHP (high hydrostatic pressure) technique.	11‐week	Scaffolds retained their shape without signs of inflammation or granulation. Tracheal stenosis and narrowing were mild	[75]
Rabbit	*n* = 20	Composite graft comprising indirectly 3D‐printed PCL and tmdECM hydrogel with hTMSC sheets	hTMSC	An indirect 3D‐printed PCL scaffold, reinforced with silicone rings, was coated with tmdECM on the lumen and layered with hTMSC sheets after a 3‐day culture. Two study groups were investigated: one with a scaffold, hTMSC sheet, and collagen, and another with a scaffold, hTMSC sheets, and tmdECM hydrogel.	8‐week	Both groups showed mild stenosis at 1 month, mainly at the anastomosis site. Group 1 developed more severe stenosis at 2 months than group 2. In group 1, a thin epithelial layer formed above granulation tissue, while group 2 exhibited complete lumen coverage with epithelial tissue at 2 months	[76]
Rabbit	*n* = 12	3D printed PCL	TBCs and autologous chondrocytes	Tracheal basal cells (TBCs) were isolated from rabbit tracheal mucosae and co‐cultured with exosomes from 3T3‐J2 cells. A 3D‐printed double‐layer PCL scaffold was designed, with autologous chondrocytes on the outer layer and TBCs on the inner layer	2‐month	Pre‐vascularized TET was transplanted into rabbits, demonstrating accelerated epithelization within 2 weeks compared to traditional methods	[77]
Rabbit	*n* = 15	Chondroitin‐sulfate‐incorporating type‐II atelocollagen (COL II/CS) scaffold	BMSCs	Incorporating chondroitin sulfate‐enhanced cartilaginous scaffolds and vascularized fibrous scaffolds to mimic the tracheal structure	N/A	Promise potential for extensive tracheal reconstruction with structural and functional similarities to the native trachea	[78]

Abbreviations: ASC, adipose‐derived stem cells; BM‐MNC, bone marrow mononuclear cell; BM, bone marrow; BMSCs, bone marrow mesenchymal stem cells; CS, cell‐seeded; FDM, fused deposition modeling; HHP, high hydrostatic pressure; hTMSC, human inferior turbinate mesenchymal stromal cell; HUVEC, human umbilical vein endothelial cell; hMSC, human mesenchymal stem cell; MSC, mesenchymal stem cell; NA, not applicable; PB, peripheral blood; PC, polycarbonate; PCL, polycaprolactone; PET, polyethylene terephthalate; PGA, polyglycolic acid; PLGA, D,L‐lactide‐co‐glycolide; PP, polypropylene; PLLA: poly(L‐lactic acid); P(LLA–CL), poly(L‐lactide‐co‐caprolactone); PU, polyurethane; PV, pre‐vascularized; RTEC, rat tracheal epithelial cell; RTC, rat tracheal chondrocyte; tmdECM, tracheal mucosa derived decellularized extracellular matrix.

### 5.3 Decellularized tracheal graft

Decellularized tracheal grafts (DTGs) have emerged as a promising TE strategy for tracheal replacement, leveraging allograft advantages such as an ideal extracellular matrix and an intact airtight structure for cellular adhesion as well as proliferation. The decellularization process involves multiple cycles of detergents and enzymes applied to harvested donor tracheal tissue over an extended period, aiming to eliminate genetic material and prevent immune reactions.^75^, ^78^ While some animal studies have shown encouraging results with observed revascularization and re‐epithelialization in specific graft areas, many attempts have faced challenges like trachea stenosis or collapse due to immunoreaction.^84^ Existing techniques struggle to achieve total removal of cellular debris or genetic material, risking adverse host responses in vivo. Striking a balance is crucial, as complete removal may compromise the airway structure's integrity as a matrix for cell proliferation and compromise physical characteristics.^85^ Despite the promise of DTGs approaches in providing a scaffold for cellular growth, their extensive processing timelines and donor‐recipient matching constraints limit their widespread applicability.^86^ However, the inherent architecture of DTGs provides a solid basis for tracheal regeneration, surpassing synthetic materials in terms of mechanical integrity. When integrated with other TE methodologies, this approach shows promise, as indicated by promising preliminary results.^87^, ^88^

### 5.4 Casting technology

Casting methodologies have emerged as pivotal techniques in the realm of TE, offering precise control over scaffold geometries within 3D structures and minimizing inherent variability.^89^ In TE applications, casting commonly involves tailored hydrogel blends, providing a high degree of customization in terms of biochemical compositions, architectural features, and mechanical properties.^90^ Notably, hydrogels have demonstrated their efficacy in promoting cellular infiltration and vascularization, showcasing successful applications in diverse areas such as skin wound healing,^91^ bone regeneration,^92^ and abdominal wall reconstruction.^93^

Examples encompass patterned 2‐hydroxyethyl methacrylate hydrogels, closely mimicking the mechanical properties of native airway,^94^ and type I collagen, hydrogels like fibrin and agarose, which have demonstrated efficacy in supporting the growth of cultured respiratory ciliated epithelium and vascular venation.^95^ While the use of hydrogels as artificial prosthetics is presently constrained, preliminary investigations in line with graft requirements have yielded promising results. However, additional studies are imperative to evaluate the mechanical integrity and feasibility of tubular constructs based on hydrogels.

## 6 ADVANCEMENTS IN CLINICAL TRANSLATION

### 6.1 Pre‐clinical evaluation of TE scaffolds

Animal studies evaluating TE tracheal substitutes have shown some success (Table 2). Many of these initiatives, however, encounter limitations due to short animal follow‐up periods, typically lasting 1–3 months.^62^, ^70^, ^71^ Initial efforts often resulted in severe inflammatory reaction, leading to serious stenosis, granulation formation, or transplant failure through infection.^49^ Ensuring airtightness and fostering vascularization are pivotal factors that substantially contribute to the survival prospects of the graft. These objectives can be accomplished through pre‐implantation strategies.^66^, ^72^ Encasing the construct in omentum not only provides a vascular network but also serves as a tissue source to maintain and seal airtightness, creating a barrier against bacterial colonization. Pre‐transplantation in the abdominal cavity facilitates omentum adherence to the substitute and the integration of the vessel.^62^ Pre‐vascularization before transplantation has demonstrated a higher animal survival rate, lower stenosis rates, and improved the re‐epithelization efficiency.^72^ Furthermore, when combined with a cell‐seeded construct, this procedure improves both cell survival and graft integration. Pre‐seeding of constructs has been observed to increase survival rates, and the integration of pre‐vascularization and pre‐seeding with chondrocytes has demonstrated a significant reduction in tracheal stenosis.^70^ Furthermore, the inclusion of pre‐seeded epithelial cells has proven effective in enhancing graft acceptance and overall transplant success when combined with mesenchymal stem cells (MSCs). This combination resulted in complete epithelial coverage of the scaffold, with no indications of distress or transplant failure observed in all cases for a duration of up to 3 months.^77^ In the most extended preclinical study to date, conducted in a canine model with a 2‐year follow‐up, a collagen‐coated nitinol frame was utilized.^62^ In this study, the implant was initially enveloped in omentum and implanted into the abdominal cavity for 3 weeks. Subsequently, it was implanted following a 2‐cm cervical tracheal resection, reporting an 80% survival rate over 1.5 years. Histological examination revealed stable epithelialization in a non‐stratified monolayer with no present secretory glands or muscular regeneration, although lumen collapse was not observed.^62^ While other studies using a similar procedure reported comparable results after 2 months, unfortunately, longer‐term follow‐ups for these studies were not reported.^65^ Several additional studies in canines have been conducted with follow‐ups for up to 1 year.^60^, ^61^

Clinical efforts of implanted synthetic TE substitutes have sparked controversy and resulted in fatalities.^96^ To understand the causes of these failures, subsequent large animal models have been utilized in pre‐clinical studies. As emphasized previously, the absence of a functional epithelium poses a substantial risk of inflammation, infection, or anastomotic fistula. Persistent inflammation observed in all cases led to lumen collapse, particularly at the proximal and distal ends of the prosthesis.^96^ Grafts seeded with MSCs exhibited a delayed onset of respiratory distress, emphasizing the significance of establishing a functional epithelium before implantation. While primarily conducted over the short term, these in vivo studies have underscored significant challenges that need addressing for the development of a viable airway substitute. The inclusion of a pre‐seeded layer seems to mitigate bacterial colonization, and pre‐vascularization enhances graft survival. However, concerns persist, particularly regarding the presence of granulation tissue in some instances.

### 6.2 Clinical evaluation of TE scaffolds

Although only a limited number of TE strategies for tracheal replacement have progressed to clinical applications, noteworthy advancements have been observed (Table 3). The initial documented success occurred in 2005,^100^ featuring a Marlex prosthesis coated with type I and II collagen. The implantation of this prosthesis demonstrated successful formation of the airway epithelium. Histological examination unveiled the successful integration of the graft into surrounding tissues, leading to the satisfactory regeneration of airway epithelium. Two years postoperatively, optimal epithelial growth was observed, confirming the successful integration of the implanted substitute. Mechanical studies further validated the patency of the airway.^100^ Subsequently, this approach was applied to a broader patient cohort, showcasing a well‐epithelialized lumen without apparent airway obstructions. This technology holds promise for treating the benign and malignant diseases of the airway.^101^ The investigation also delved into the potential synergy of this technology with growth factor delivery to enhance graft performance and achieve optimal regeneration universally. The hypothesis entailed the addition of basic fibroblast growth factor (bFGF) to the cartilage defect, with the goal of enhancing vascular formation and preserving tracheal patency. Nevertheless, the use of bFGF might be restricted in cases involving malignant tumor populations due to an elevated risk of recurrence.^102^

**TABLE 3:** Transitioning TE and non‐TE strategies for tracheal reconstruction and regeneration in human clinical trials.

Patients	Technology	Graft	Cell type	Procedure	Clinical outcome	References
*TE strategies*						
26‐year‐old male	Decellularization	Autologous cell‐populated decellularized porcine jejunum	Recipient's mvECs and skMCs	Patient with extensive tracheal and esophageal defect underwent reconstruction using porcine cell‐free vascularized scaffolds. The scaffolds were obtained through a decellularization process and seeded for re‐endothelialization with recipient cells before implantation into the 5 × 2 cm defect. Characterization of the construct was performed to ensure safety and optimal performance prior to implantation	The postoperative phase proceeded without complications, and the transplanted bioengineered construct exhibited complete integration, showcasing a fully functional respiratory epithelium lining the airway. Notably, there was no evidence of tissue scar formation or dedifferentiation	[97]
12‐year‐old male	Decellularization	Decellularized human tracheal graft with autologous stem‐cell implanted	Hematopoietic stem cells expressing CD34 or CD45wk, along with mesenchymal stem cells expressing CD73+, CD90+, CD105+, CD117+, or CD45+ in the recipient	The decellularized cadaveric trachea was infused with the recipient's stem cell suspension, followed by implantation with a PDO stent. The construct was further loaded with hrEPO, G‐CSF, and TGF‐β	Vascularization of the graft was observed 1 week after surgery, with epithelium restoration becoming apparent only 1 year post‐implantation. Biomechanical strength reached optimal levels at 18 months, and functional airway was confirmed at the *2*‐year follow‐up	[98]
15‐year‐old female	Decellularization	Decellularized human tracheal graft with autologous stem‐cell implanted	Recipient's bone marrow‐derived mesenchymal stem cells (BM‐MSCs) and epithelial cells derived from the nasal region	The decellularized cadaveric trachea was pre‐seeded in a custom‐made bioreactor in vitro prior to implantation	Thirteen days post‐surgery, tracheal graft narrowing was observed, causing compromised ventilation and respiratory arrest, ultimately resulting in cerebral hypoxic injury and edema	[99]
78‐year‐old female	Artificial scaffold	Marlex mesh tube coated with a sponge made from collagen type I and III (dermal atelocollagen)	N/A	The structure was implanted and infused with the recipient's autologous venous blood to ensure air‐tightness, water‐tightness, and release of endogenous factors	The engineered construct facilitated epithelial growth 2 months post‐surgery achieved proper epithelialization at 7 months, and was fully covered with respiratory epithelium after 20 months. Continuous epithelialization over the trachea occurred for 2 years without complications	[100]
Four patients (59–78 years; male, *n* = 2)	Artificial scaffold	Marlex mesh tube coated with a sponge made from collagen type I and III (dermal atelocollagen)	N/A	The implantation involved injecting the construct with autologous venous blood	During the observation period spanning 8–34 months, the construct exhibited substantial epithelialization without any obstructions	[101]
Three patients (39–71 years; male, *n* = 2)	Artificial scaffold	Marlex mesh tube coated with a sponge made from collagen type I and III (dermal atelocollagen)	N/A	Two‐stage operation involved the resection of stenotic regions and subsequent implantation of the construct, followed by the delivery of venous blood and b‐FGF to address the cartilage defect	All patients exhibited improved respiratory function post‐implantation, experiencing no discomfort in their daily activities. Sufficient air space in the trachea was observed 6 months after the procedure	[102]
2‐month‐old infant	3D printing scaffold	PCL	N/A	An individually tailored and manufactured resorbable 3D‐printed airway splint was implanted, with an anticipated complete resorption expected within a 3‐year timeframe.	One year post‐surgery, a patent left mainstem bronchus was observed, and no unexpected issues related to the splint were encountered	[103]
Three patients (3–16 months; male, *n* = 3)	3D printing scaffold	PCL	N/A	Scaffold were tailored to individual patient requirements using CT imaging and CAD modeling software	All patients exhibited no signs of airway disease, indicating sustained growth of the primary airways	[66]
46‐year‐old female	3D printing scaffold	PCL	N/A	A 3D‐printed splint, designed with the aid of CT imaging and modeling software, was implanted and enveloped in an artificial pleural patch	Enhanced respiratory and physical strength were observed in a 3‐month follow‐up, with no discernible adverse reactions or toxic effects	[104]
14‐year‐old female	3D printing scaffold	PEKK	N/A	A PEKK splint was custom‐designed using CT imaging and modeling software	Achievement of tracheal patency with the patient remaining asymptomatic, and no subsequent complications or hospitalizations were documented	[105]
15 patients (3–25 months; male, *n* = 6)	3D printing scaffold	PCL	N/A	3D‐printed split designed through CT imaging and modeling software	No patients necessitated splint removal or re‐operation. Three mortalities were recorded during the 8.5‐month follow‐up	[65]
*Non‐TE strategies*						
43‐year‐old female	Autograft	Forearm free flap reinforced with an Ultraflex stent was utilized	N/A	A free flap was harvested and enfolded around a stent following implantation to address a 6 cm tracheal defect	Passed away 16 months post‐procedure due to pre‐existing conditions	[106]
63‐year‐old female	Autograft	Forearm‐free flap reinforced with external mesh support	N/A	A radial forearm fasciocutaneous flap was employed in combination with a Hemashield vascular graft and PolyMax resorbable mesh	The patient remains asymptomatic at the 6‐month mark, having resumed normal activities. A follow‐up bronchoscopy revealed slight migration but a healed flap without obstruction	[107]
16 patients (37–68 years; male, *n* = 9)	Autograft	Rib cartilage and a fascial skin pad	N/A	Autologous cartilage segments were incorporated into a forearm skin pad, creating a construct that was enveloped around a silicone tube for suturing and subsequently implanted within the defect	Three fatalities occurred post‐surgery, attributed to lung infections, acute respiratory distress syndrome, and myocardial infarction. A long‐term follow‐up revealed a 65% survival rate	[108]
21‐year‐old male	Allograft	Donor cadaveric tracheal graft	N/A	A cadaveric trachea with preserved blood supply underwent heterotopic implantation into the sternocleidomastoid muscle for 3 weeks. Subsequently, it was orthopically implanted into the tracheal defect along with a vascularized muscular segment of the sternocleidomastoid	The seamless incorporation of the tracheal graft, coupled with sustained functionality over a period of 9 weeks, is achieved with an absence of discernible signs of rejection, ischemia, or infection	[109]
24‐year‐old female	Allograft	Donor cadaveric tracheal graft	N/A	The allograft was surgically implanted and enveloped with omentum, while the patient concurrently underwent immunosuppressive therapy	The postoperative course was uneventful, with no signs of graft rejection, necrosis, or infections. A silicon endoprosthesis was required, but signs of rejection diminished, and a one‐year follow‐up confirmed the patient's continued survival with a restored tracheal lumen	[110]
Four patients (17–64 years; male, *n* = 3)	Allograft	Donor cadaveric tracheal graft	Recipient's buccal mucosa and/or engrafted recipient cells	Decellularized tracheas were surgically implanted in the forearm and augmented with either buccal mucosa grafts or enveloped in forearm fascia to enhance vascularization	Tracheal necrosis ensued upon immunosuppression withdrawal, causing partial allotransplant loss in three patients due to insufficient vascularization. However, one patient, implementing additional recipient cell repopulation strategies, achieved a vascularized allotransplant with restored normal airways within 6 months post‐transplantation.	[111]
68‐year‐old male	Autograft	Aorta autograft with Dumon stent	N/A	A 7 cm autograft from the abdominal aorta was harvested and substituted with a Dracon graft. The aortic graft was implanted along with a silicone Dumon stent to prevent injury to the aortic wall	Granulation tissue formation triggered acute respiratory distress syndrome (ARDS), prompting intervention with an extra tracheal stent. Subsequently, stent removal ensued due to migration, although no evidence of airway collapse was observed. Regrettably, the patient's demise occurred due to septic shock induced by complications, including pneumonia, respiratory distress, and pneumothorax	[112]
Six patients (17–52 years; male, *n* = 5)	Allograft	Aortic allografts with Dumon stent	N/A	Aortic allografts were enveloped with highly vascularized pectoral muscle or thymopericardial fat flaps and implanted with the assistance of a silicone stent	Complete resection was successfully accomplished in 83% of patients, with outcomes marked by minimal major morbidity, absence of fistulas, and uneventful recoveries. All grafts exhibited satisfactory vascularization, and four patients remain disease‐free	[113]
78‐year‐old male	Allograft	Aortic allografts with Dumon stent	N/A	The lung cancer was resected, and a stent‐supported graft was surgically implanted	One year post‐procedure, the re‐implanted lobe exhibited optimal function, and the patient regained baseline activity levels, enjoying a satisfactory quality of life	[114]
20 patients (24–79 years; male, *n* = 13)	Allograft	Aortic allografts with Dumon stent	N/A	Radial tumor resections were conducted, followed by the surgical implantation of a stent‐supported graft, which was circumferentially covered with a local muscle flap	A 90‐day patient follow‐up revealed a 5% mortality rate, with no adverse effects observed from the surgical technique. A 76.5% survival rate was documented at a median follow‐up of 3 years and 11 months, accompanied by evidence of cartilage and respiratory epithelium regeneration	[115]
35 patients (24–79 years; male, *n* = 19)	Allograft	Aortic allografts with Dumon stent	N/A	Radial tumor resections were conducted, followed by the surgical implantation of a stent‐supported graft, which was circumferentially covered with a local muscle flap	The 30‐day postoperative mortality and morbidity rates stood at 2.9% and 22.9%, respectively. A median follow‐up of 29.5 months revealed 27 surviving patients, with 28.6% achieving stent‐free survival. The actuarial 2‐ and 5‐year survival rates were 88% and 75%, respectively	[116]

Abbreviations: BM‐MNC, bone marrow mononuclear cell; b‐FGF, basic fibroblast growth factor; CAD, computer‐aided design; CT, computed tomography; G‐CSF, granulocyte‐colony stimulating factor; hrEPO, human recombinant erythropoietin; MSC, mesenchymal stem cells; mvEC, microvascular endothelial cell; N/A, not applicable; PCL, polycaprolactone; PDO, polydioxanone; PEKK, polyetherketoneketone; PP, polypropylene; skMC, skeletal muscle cell; TGF‐β, transforming growth factor‐β; TBM, tracheobronchomalacia; 3DP, 3D printing‐2.

In 2010, the inaugural successful surgical implantation of DTGs took place, involving the transplantation of a decellularized cervical tracheal graft seeded with buccal mucosa.^109^, ^110^ Although this approach was extended to a cohort of four patients, graft poor graft vascularization and necrosis still posed challenges in the clinical trial's outcome.^101^ In a different series of attempts, cadaveric tracheas underwent decellularization and pre‐seeding with the recipient's MSCs and growth factors before implantation. The objective was to ensure substitute re‐vascularization and promote chondrogenesis. The results showed varying outcomes, including tracheal narrowing. However, a particular DTG successfully regenerated epithelium around the damaged section without complications during a 4‐year follow‐up.^98^, ^99^ While some TE approaches have shown promise, further extensive follow‐up period research works involving a larger cohort are imperative to establish this technology as a viable alternative for tracheal reconstruction.

As previously mentioned, 3D printing technology has emerged as a potential approach for addressing airway lesions in pediatric patients, with recent FDA approval for 3D‐printed airway splints.^1^ The application of a 3D‐printed splint to treat a serious patient of TBM was first published in the beginning of the 20th century^103^ and has since been extended to a broader population, showing low general mortalities.^104^, ^105^ However, the utilization of 3D‐printed tracheal splints has primarily been limited to pediatric populations with TBM, leveraging guided airway growth during early developmental stages to facilitate natural resolution of TBM. Apart from cases involving pediatric cases, there is only one documented instance of using a 3D‐printed airway splint in an adult, demonstrating sustained airway patency 3 months post‐procedure. Ongoing clinical trials are also exploring custom‐made airway stents. These stents are meticulously crafted using patient‐specific sacrificial molds 3D‐printed from CT scanning, resulting in personalized tracheal prosthetics made from silicone elastomer. Several patients in clinical applications have reported an improved quality of life with no observed complications. While this personalized approach shows promise, long‐term results are still pending. Despite early successful attempts to use 3D printing for tracheal stenosis and malignancies, there is a crucial need to extend follow‐up studies to assess splint resorption. Additionally, establishing optimal designs and validated manufacturing processes is vital to ensure safety and efficacy in future clinical application attempts.^117^

The first step in the successful development of a TE tracheal substitute involves a thorough characterization of the proposed construct. This encompasses the assessment of material performance, degradation, mechanical strength, and flexibility. Moreover, the manufacturing process ideally allows for the production of individualized constructs tailored to specific patient needs, incorporating antibacterial, antiproliferative, antitussive, and non‐migrating properties.^2^, ^3^ A limitation in tissue‐engineered substitutes is the absence of consistent and relevant data on airway biomechanics, standardized tests for evaluating mechanical properties, and a diverse range of mechanical values for potential grafts.^118^ Therefore, there is a need to establish a standardized procedure for evaluating mechanical strength to better comprehend the requirements of TE material. This standardized procedure should also account for variations in airway biomechanics among patients of diverse genders or age groups.^116^ Various scaffolds have been devised to support epithelialization in grafts, as a mature pseudostratified epithelial lining is crucial to prevent tissue outgrowth, granulation, and stenosis.^119^ Various strategies have been explored in both laboratory settings and living organisms. However, the absence of prolonged evaluations and the occurrence of granulation and inflammatory reactions after transplantation have impeded further progress. This underscores the need for enhanced TE approaches that facilitate comprehensive and functional re‐epithelialization early in the graft development process.^117^ Vascularization of TE scaffolds remains a critical challenge that must be addressed for stents to progress into clinical applications. It is widely acknowledged that vascularization is imperative to achieve a functional substitute, as the absence of vascular formation inevitably leads to poor outcomes, no benefit for the patient, and high morbidity.^120^ Although several procedures reveal promise in implant revascularization, further long‐term assessment of patients and adequate trials are necessary to demonstrate the suitability of these strategies for airway reconstruction.^100^, ^101^

In summary, the restricted TE strategies for tracheal reconstruction that have transitioned into clinical applications have provided significant insights into the crucial requirements for a successful airway substitute. Considering the intricate anatomy of the trachea, a myriad of challenging properties must be collectively addressed. Insights from both pre‐clinical and clinical trials have enhanced our understanding of the advantages and challenges associated with specific techniques and methods.

### 6.3 Clinical evaluation of non‐TE technologies

#### 6.3.1 Artificial prosthesis

Following initial challenges in treating long‐segment airway lesions, attention shifted towards replacing the trachea using polymer prostheses in solid or porous forms. The Neville prosthesis, a solid siloxane polymer prosthesis, demonstrated success in animal studies but faced challenges in humans, leading to stenosis and graft failure.^121^, ^122^ The mismatch in mechanical properties, rigidity causing erosion of adjacent blood vessels, and the inability to integrate within surrounding tissue resulted in dislodgement during coughing and airway obstruction.^123^ Other solid prostheses, including polyethylene (PE),^124^ stainless‐steel wire,^125^ silicone stent,^126^ and tantalum,^127^ have also been explored as alternatives in tracheal reconstruction.

Recognizing issues with solid prostheses, research turned to porous alternatives to facilitate better tissue formation and structure regeneration. A notable development in porous prostheses occurred with the successful development of Marlex mesh prosthesis, made from high‐density PE and polypropylene. In an animal study, it demonstrated adequate graft survival and patency during the 16‐months follow‐up.^128^ However, its human application was disappointing, marked by erosion of surrounding blood vessels and lumen collapse.^129^ The Marlex prosthesis highlighted the significance of respiratory epithelium growth on the inner lumen, serving as a protective barrier against inhaled foreign bodies.^130^ Other attempts at porous prostheses included PE, silicone, dermal grafts,^66^ wire mesh,^131^ and biomedical engineering tubes, but these yielded similarly unsatisfactory results.^132^

Current treatment for tracheobronchial complications often involves commercially available stents, such as the silicone‐based self‐expanding PolyFlex™,^133^ the Ultraflex™,^134^ Gold Studded Stents® and Novatech's silicone Dumon®.^135^ Clinical application of silicone stents has generally improved symptoms and quality of life. Despite being well‐tolerated in most cases, long‐term follow‐ups have revealed significant drawbacks, including granulation tissue obstruction, stent migration, excessive immune response, infections, mucus retention, and lumen collapse, as documented in researche with extended follow‐up periods.^136^, ^137^

#### 6.3.2 Aortic allograft

Allograft implantation for long‐segment tracheal lesions has mainly concentrated on two donor tissue sources: tracheas and aortas. Evaluations of the viability of fresh and cryopreserved stented aortic grafts as a potential substitute for tracheal reconstruction have verified newly formed cartilage and respiratory epithelium regeneration in many animal models. However, some pre‐clinical studies have reported unsatisfied cartilage regeneration.^138^ Despite these promising findings in preclinical models, clinical attempts to utilize stented aortic grafts as donor tissues for airway replacement have raised notable concerns. In a 2006 case, a 68‐year‐old male experienced severe complications and ultimately succumbed to the procedure involving aortic allograft transplantation.^112^ In another instance, a 78‐year‐old male was supported with a stent after the implantation of a cryopreserved and stented aortic allograft. However, this case exhibited no cartilage formation, accompanied by a decrease in forced expiratory volume.^114^ Similar concerns were observed in a clinical cohort study involving six patients who received cryopreserved descending aortas that were silicone‐stented and subcutaneously implanted for pre‐vascularization. Long‐term follow‐ups revealed minor complications, including stent migration, blood vessel erosion, and even tracheoesophageal fistulas in some cases. Furthermore, biopsied tissues showed no evidence of newly formed cartilage or respiratory epithelium regeneration.^139^

The TRITON‐1 study (Clinicaltrials.gov Identifier: NCT04263129), led by Martinod's group from October 2009 to October 2021, involved 35 patients undergoing trachea or bronchus reconstruction for both benign and malignant lesions. The overall hospital mortality rate was 2.9%. With a median follow‐up of 2.5 years, 77.1% of patients remained in good health condition, with no reported deaths directly attributed to the implanted grafts. Scaffold‐related granulomas requiring bronchoscopic treatment occurred in 52.9% of patients. Furthermore, 28.6% of patients achieved stent‐free survival, with a 5‐year survival rate of 75%.^116^

#### 6.3.3 Tracheal graft

The inaugural human tracheal transplantation, conducted in 1979, demonstrated no graft rejection or major complications during a 2‐month follow‐up.^109^ In 1990, another early success involved a one‐stage primary tracheal replacement with a silicone stent and extensive immunosuppressive therapy, with no reported issues in a follow‐up duration of 2 years.^110^ Despite these early achievements, subsequent attempts at trachea transplantation encountered limited clinical success.^140^ Recent advancements by Delaere et al.^141^ introduced a two‐step procedure, involving the revascularization of the tracheal graft in the patient's forearm before implantation.^142^ Although tissue necrosis occurred in some cohort studies, the technology remains promising.^140^

Allograft transplantation, providing a structurally sound analogue, exhibits variable success. Successful cases often involve long‐term immunosuppressive therapy, which may be unsuitable for malignant cases. Allografts, lacking adequate mechanical properties and cartilage regeneration, usually necessitate stent support.^140^ However, stents bring new challenges, including anastomotic fistula, erosion of adjacent tissue and blood vessels, infection, and granulation formation. Poor re‐vascularization of grafts requires implantation into a second surgical site, heightening hospitalization risks and infection.^140^ Despite limited success with traditional organ implantation, past attempts provide insights into the requirements for successful tracheal grafts, steering current research toward overcoming these challenges using TE strategies.

Clinical exploration of tracheal transplantation with autologous tissue involves a two‐step operation procedure. Tracheal autografts involve harvesting cartilage from ribs, encasing it within a forearm‐derived fascial skin pad, and wrapping it around a temporary silicone scaffold for transplantation. Preserving the radial artery and skin pad veins is vital for post‐transplant conduit revascularization. This technique eliminates the need for immunosuppressants, as no synthetic materials are introduced.^143^ However, challenges exist: autografts lack a respiratory epithelial layer and depend on healthy cartilage and respiratory function, posing risks like secretion obstruction and cartilage fracture leading to implant failure.^143^, ^144^

## 7 CONCLUSION

Over the past decade, TE strategies have emerged for tracheal tissue regeneration, but their clinical application has been limited and small‐scale. Challenges such as poor mechanical properties, insufficient vascularization, and inadequate re‐epithelialization have hindered graft performance. Long‐term follow‐up assessments in both pre‐clinical and clinical studies are lacking, essential for evaluating feasibility. A successful airway reconstruction construct must have the necessary mechanical properties and support the formation of vascular capillaries and respiratory epithelium to prevent complications like granulation formation, contamination, tissue necrosis, fistula, and lumen collapse. TE in airway reconstruction, highlighted by promising prototypes and clinical trials, provides insights into essential properties for a successful tracheal substitute. Addressing challenges in mechanical properties, epithelialization, and vascularization could revolutionize the clinical approach to long‐segment tracheal lesions.

## AUTHOR CONTRIBUTIONS

**Shixiong Wei:** Data curation; writing – original draft. **Yiyuan Zhang:** Software; supervision; writing – review and editing. **Feixiang Luo:** Data curation; formal analysis; funding acquisition. **Kexing Duan:** Methodology; resources; software; validation. **Mingqian Li:** Writing – review and editing. **Guoyue Lv:** Conceptualization; writing – review and editing.

## CONFLICT OF INTEREST STATEMENT

All authors confirm that there is no potential conflict of interest.

## Data Availability

The datasets generated and/or analyzed during the current study are available from the corresponding author on reasonable request.
